# Maternal Prepregnancy Overweight: Associations With Maternal and Offspring Weight 4–7 Years Postpartum

**DOI:** 10.1155/jdr/9989579

**Published:** 2026-02-05

**Authors:** Ning Yuan, Dan Zhao, Xiumei Xu, Xiaomei Zhang

**Affiliations:** ^1^ Department of Endocrinology, Peking University International Hospital, Beijing, China, pku.edu.cn

**Keywords:** maternal and offspring overweight and obesity, prepregnancy overweight and obesity

## Abstract

**Background:**

Studies have shown that prepregnancy overweight and obesity can increase the risk of gestational complications. However, research on the medium‐ to short‐term impact on mothers and their offspring is limited. This study is aimed at investigating the association between prepregnancy overweight and obesity and subsequent weight issues in mothers and their children, specifically focusing on the period spanning 4–7 years postpartum.

**Methods:**

This prospective cohort study included 112 mother–child pairs recruited from Peking University International Hospital between 2017 and 2019. Anthropometric and metabolic parameters were assessed in mothers and their offspring 4–7 years postpartum. Mothers also underwent an oral glucose tolerance test (OGTT) and assays for lipid, inflammatory, and adipokine markers. Based on prepregnancy body mass index (BMI), participants were classified into an overweight and obese group (*n* = 28) or an underweight and normal weight group (*n* = 84).

**Results:**

At 4–7 years postpartum, a higher proportion of mothers with prepregnancy overweight and obesity remained overweight (39.29%) or obese (39.29%), compared to mothers who were underweight and normal weight before pregnancy (13.10% overweight, 0% obese). Among offspring, the prevalence of obesity was higher in children of the overweight and obese maternal group (17.86% vs. 7.14%). After adjusting for various factors such as parity, gestational age, gestational weight gain, and gestational diabetes mellitus (GDM), logistic regression analysis indicated that prepregnancy overweight and obesity were strongly associated with maternal overweight and obesity at follow‐up (OR = 30.70, 95% CI: 8.69–108.51, *p* < 0.01) but not with offspring overweight and obesity (OR = 1.49, 95% CI: 0.54–4.06, *p* = 0.440).

**Conclusions:**

This study demonstrates a strong association between prepregnancy overweight and obesity and lasting weight issues 4–7 years postpartum, underscoring the importance of preconception weight management as a key preventive health strategy.

## 1. Introduction

The global obesity crisis has garnered immense attention, and China is no exception. With the rapid economic growth and the subsequent improvement in living standards, the obesity problem has become increasingly prominent in the country. Epidemiological surveys have consistently shown a rising prevalence of overweight and obesity among Chinese adults. A recent study involving 15.8 million adults found that, according to the Chinese body mass index (BMI) classification, 34.8% of adults were overweight and 14.1% were obese [1]. Moreover, this trend is not confined to adults; childhood and adolescent obesity rates have also surged. According to the latest national prevalence estimates for 2015–2019, 6.8% of children under 6 years old were overweight, while 3.6% were obese. Among children and adolescents aged 6–17 years, the prevalence of overweight and obesity was 11.1% and 7.9%, respectively [2]. With obesity rates escalating globally and within China and the rising trend of delayed childbearing, the issue of prepregnancy overweight and obesity has come to the forefront. Several studies have indicated that prepregnancy overweight and obesity increase the risk of various pregnancy complications, such as stillbirth, gestational diabetes mellitus (GDM), gestational hypertension or preeclampsia, cesarean section, large for gestational age (LGA) infants, and preterm birth [3–7]. These findings highlight the critical need for addressing prepregnancy overweight and obesity to improve maternal and child health outcomes.

Prepregnancy overweight and obesity have detrimental effects on both perinatal outcomes and the long‐term health of mothers and their offspring. Prior research has consistently shown that these conditions can lead to postpartum weight retention (PPWR), characterized by the persistence of excess weight after childbirth, and abnormalities in various metabolic indicators, which increase the risk of postpartum overweight and obesity for mothers [8, 9]. This highlights the ongoing burden that prepregnancy weight status places on maternal health. Furthermore, the link between prepregnancy overweight and obesity and the long‐term health of offspring is well documented. Children born to mothers with these conditions are at a higher risk of developing obesity and metabolic abnormalities later in life [10, 11]. The mechanisms behind this influence are multifaceted and complex, involving alterations in fetal adipose tissue and endocrine system development, as well as epigenetic modifications that can be transmitted to offspring, affecting gene expression patterns and contributing to obesity and related comorbidities. Given the profound and long‐lasting implications of prepregnancy overweight and obesity, addressing this issue before conception is crucial for promoting maternal and child health. Therefore, further research is needed to elucidate the precise mechanisms underlying these associations and to develop effective prevention and management strategies specifically tailored to this population.

Despite numerous studies examining the links between prepregnancy weight, postpartum weight changes, and offspring obesity, inconsistencies in their findings persist. Additionally, most studies have primarily focused on the perinatal period, with limited attention paid to the middle to late postpartum stages. The present study is aimed at analyzing the influence of prepregnancy overweight and obesity on maternal and offspring overweight and obesity status 4–7 years postpartum. By doing so, we hope to elucidate the association between prepregnancy overweight and obesity and maternal and offspring obesity. Our ultimate goal is to provide a scientific foundation for the development of effective prevention and intervention strategies for prepregnancy weight management.

## 2. Materials and Methods

### 2.1. Study Population

This study established a new prospective mother–child cohort by extending the follow‐up of an existing birth cohort, which originally comprised women who delivered at Peking University International Hospital between 2017 and 2019. Mother–child pairs potentially eligible for the extended follow‐up were first identified from the original cohort database based on initial criteria including singleton pregnancy, maternal age between 20 and 45 years at delivery, and availability of contact information. These potentially eligible individuals were then contacted via recruitment advertisements or telephone and invited to participate in the current extended follow‐up study, with visits scheduled when the child was between 4 and 7 years of age. For inclusion in the present extended cohort, offspring were required to be between 4 and 7 years old at follow‐up. Exclusion criteria consisted of multiple pregnancies, maternal history of Type 1 or Type 2 diabetes, GDM with insulin treatment during pregnancy, diagnosis of rheumatic immune diseases, severe liver or kidney dysfunction, and long‐term use of antidepressants or steroid medications. After assessment of eligibility and completion of the follow‐up visit, a total of 112 mother–child dyads were ultimately included in the present study, with both mothers and their offspring followed up from 4 to 7 years postpartum.

### 2.2. Definitions and Methods

**Figure 1:**
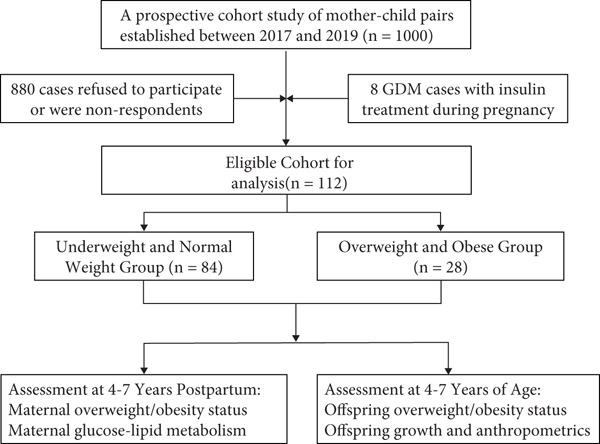
Flowchart of study participants. GDM, gestational diabetes mellitus.

Each participant′s BMI was calculated by body weight in kilograms divided by the square of height in meters. According to the Chinese BMI classification [1, 2], underweight and normal weight were defined as < 24 kg/m^2^, overweight as 24–< 28 kg/m^2^, and obesity as ≥ 28 kg/m^2^. The definitions of childhood overweight and obesity are based on the threshold values of BMI for children aged 4–7 years, as outlined in the article “Body Mass Index Growth Curves for Chinese Children and Adolescents Aged 0 to 18 Years” [12]. Based on their prepregnancy BMI levels, the participants in this study were divided into two groups: the overweight and obese group (*n* = 28) and the underweight and normal weight group (*n* = 84) (Figure 1).

Maternal prepregnancy weight was obtained by maternal recall at the first antenatal visit. Maternal height and weight at that first visit were objectively measured and recorded in the prenatal medical record. Prepregnancy BMI was calculated using the recalled prepregnancy weight and the objectively measured height. The height and weight of all children at the 4–7‐year follow‐up were assessed through direct physical measurement during the study visit. These measurements were taken by trained research nurses or physicians using calibrated stadiometers and digital scales, following standardized protocols.

All enrolled women provided informed consent. At baseline, they completed a questionnaire covering demographic information, obstetric and gynecological history, and past medical conditions (including thyroid disorders and diabetes), as well as maternal age, parity, and self‐reported prepregnancy weight and height. Data pertaining to pregnancy and delivery were obtained through retrospective review of medical records. These included gestational weight gain (GWG), results of the oral glucose tolerance test (OGTT), gestational age at delivery, and the infant′s sex. Neonatal outcomes were also extracted, encompassing birth weight, length, neonatal sex, head circumference, Apgar scores, and the presence of conditions such as prematurity, hyperbilirubinemia, respiratory distress, asphyxia, hypoglycemia, and hypoxic encephalopathy.

### 2.3. Follow‐Up Index

The postpartum period, lasting from 4 to 7 years, involved monitoring various health indicators such as blood pressure, maternal anthropometric measurements including height, weight, waist and hip circumference, triceps skinfold thickness (TSF), and body fat percentage. Additionally, glycemic metabolism was assessed through an OGTT, fasting and 120‐min insulin levels, insulin resistance index (HOMA‐IR), and fasting and 120‐min C‐peptide, as well as HbA1c levels. These comprehensive evaluations were aimed at providing a thorough understanding of maternal health during the postpartum period.

The analysis of lipid metabolism involved assessing various indicators including total cholesterol (TC), triglycerides (TG), high‐density lipoprotein cholesterol (HDL‐C), low‐density lipoprotein cholesterol (LDL‐C), TC/HDL‐C ratio, small dense low‐density lipoprotein (sd‐LDL), apolipoprotein A1 (ApoA1), apolipoprotein *β* (Apo*β*), lipoprotein(a), and free fatty acids (FFAs). In addition to lipid markers, inflammatory markers such as interleukin‐6 (IL‐6), interleukin‐8 (IL‐8), interleukin‐2 (IL‐2), interleukin‐1*β* (IL‐1*β*), tumor necrosis factor alpha (TNF‐*α*), tumor necrosis factor beta (TNF‐*β*), and growth differentiation factor 15 (GDF15) were also considered, along with the adipokines adiponectin and leptin. Waist‐to‐hip ratio (WHR), Conicity index (*C*‐index), visceral adiposity index (VAI), and lipid accumulation product (LAP) were calculated to gain insights into adiposity. Moreover, blood pressure and anthropometric measurements were taken in children aged 4–7, including height, weight, waist circumference, hip circumference, and TSF, providing comprehensive data on their health status.

### 2.4. Clinical Care Setting

All participants received routine, guideline‐based antenatal care at the study hospital. Standard practice included the provision of a personalized weight gain chart with targets based on prepregnancy BMI according to national guidelines. Maternal weight was monitored at each prenatal visit, and brief lifestyle counseling was provided by the obstetrician or midwife. Additionally, women had access to “Pregnant Women′s School” sessions covering nutrition and exercise. If weight gain deviated from the recommended range, a referral was made to a clinical dietitian for individualized management.

### 2.5. Outcome Parameters

This study focused on exploring the long‐term impact of prepregnancy overweight and obesity on both maternal and offspring health 4–7 years after childbirth. The primary objective was to analyze the association between prepregnancy weight status and the likelihood of maternal and offspring overweight and obesity during this period. Additionally, secondary outcomes included examining how prepregnancy BMI influenced postpartum metabolic health, inflammation levels, and indicators of abdominal obesity in both mothers and their children.

### 2.6. Selection of Covariates

Covariates for adjustment were selected a priori based on their established or plausible role as confounders in the association between prepregnancy BMI and maternal and offspring outcomes, informed by previous literature [13–15]. Parity, gestational age, and GDM status were adjusted for as common causes of exposure and outcomes. Due to its plausible mediation, GWG was analyzed in two ways: Models without GWG estimated the total effect, and models with GWG estimated the direct effect.

### 2.7. Ethics Approval and Consent to Participate

The research was conducted in adherence to the ethical principles outlined in the Helsinki Declaration and adhered to the STROBE guidelines for reporting observational studies. Prior to the initiation of the study, ethical approval was granted by the Ethics Committee of Peking University International Hospital. Furthermore, written informed consent was meticulously obtained from each participant, ensuring their voluntary participation and comprehension of the study objectives, prior to enrollment and sample collection. For the offspring participants, the written informed consent was procured from their legal guardians, ensuring the protection of their rights and welfare throughout the research process.

### 2.8. Sample Size Calculation

This research project delves into a cohort study that seeks to classify individuals into two distinct groups based on their weight status: the overweight and obese group and the underweight and normal weight group. The main objective of this study is to investigate the relationship between prepregnancy overweight and obesity with maternal and offspring overweight and obesity 4–7 years after giving birth. Drawing on previous research [16] that highlighted a transition rate of 32.95% among overweight women and 2.61% among normal weight women toward postpartum obesity in the general population, the study set statistical parameters at an alpha level of 0.05 (two‐tailed) and a beta (*β*) level of 0.10 to ensure the reliability of the results. Sample size calculations were meticulously conducted using the PASS 11 for both the exposed and nonexposed groups, maintaining a ratio of 1:4. The analysis indicated a total sample size requirement of 50 subjects, with 10 individuals in the exposed group and 40 in the nonexposed group. To account for potential attrition during the study, the final sample size was conservatively adjusted to 56 subjects, ensuring the robustness of the findings. By rigorously adhering to these methodological procedures, the study is aimed at providing valuable insights into the long‐term effects of prepregnancy weight status on maternal and offspring health, ultimately contributing to the existing body of knowledge on this critical issue.

### 2.9. Statistical Analysis

This study utilized the R statistical package and Free Statistical Software Version 1.7.1 for all statistical analyses. Mean and standard deviation were calculated for normally distributed data, while median and interquartile range (IQR) were used for nonnormally distributed data. Categorical variables were presented as counts and percentages. Group comparisons were made using analysis of variance (ANOVA) for normally distributed data and nonparametric tests for nonnormally distributed data. The choice between the chi‐square test and Fisher′s exact test was based on data appropriateness for categorical variables. The study was aimed at exploring the relationship between prepregnancy BMI and various metabolic, inflammatory, and anthropometric factors in mothers and their offspring aged 4–7 years postpartum through multiple regression analysis. Results were reported as odds ratios (ORs) with 95% confidence intervals (CIs). Additionally, logistic regression was used to examine the association between prepregnancy overweight and obesity and the risk of overweight and obesity in both mothers and offspring. All statistical tests were performed with a two‐sided approach and a significance level (*α*) of 0.05. A *p* value lower than 0.05 was considered statistically significant, ensuring the credibility and validity of the study findings. Through these rigorous statistical methods, valuable insights into the impact of prepregnancy BMI on postpartum health outcomes were obtained, shedding light on potential risk factors for overweight and obesity in mothers and their children.

## 3. Results

A total of 17.86% (*n* = 20) of the maternal participants were diagnosed with overweight, and 7.14% (*n* = 8) were affected by obesity prior to pregnancy, based on their prepregnancy BMI classification. In the 4–7 years postpartum period, the prevalence of overweight among maternal participants in the underweight and normal weight group was 13.10%, with a 0% obesity rate. Conversely, in the overweight and obese group, the proportions were 39.29% for overweight and 39.29% for obesity, indicating a significant difference in the rates of overweight and obesity between these two groups. Among the offspring aged 4–7 years, the underweight and normal weight maternal group had a 16.67% overweight rate and a 7.14% obesity rate, whereas the overweight and obese maternal group exhibited a 14.29% overweight rate and a 17.86% obesity rate among their offspring. However, no statistically significant difference was observed in the proportions of overweight and obese offspring between these two maternal groups (Figure 2).

Figure 2The prevalence of maternal and offspring overweight and obesity 4–7 years postpartum in the overweight and obese group and the underweight and normal weight group.

**Figure:**
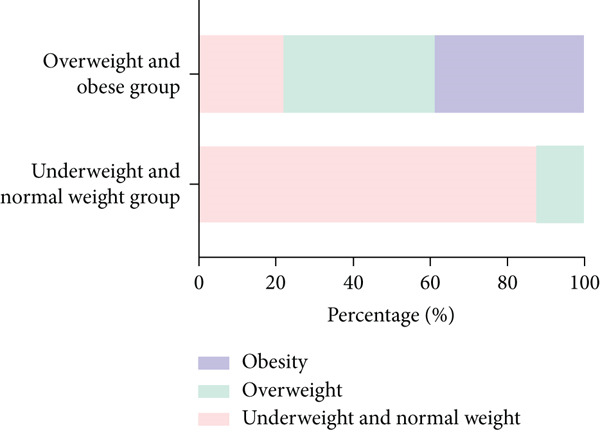
(a) The prevalence of maternal overweight and obesity 4–7 years postpartum

**Figure:**
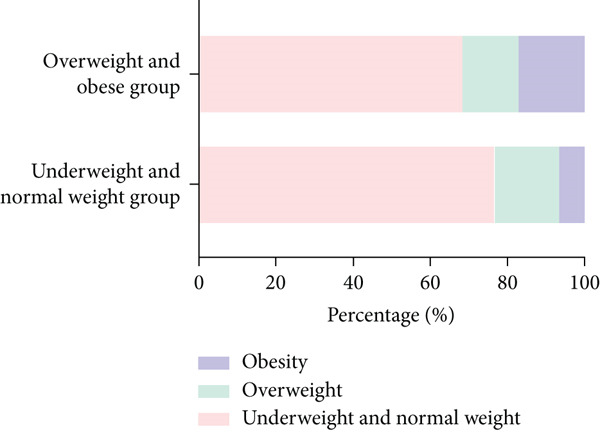
(b) The prevalence of offspring overweight and obesity 4–7 years postpartum

The maternal and offspring characteristics of the study participants during pregnancy and the perinatal period are presented in Table 1. The proportion of mothers in the underweight and normal weight group who gained more than 11 kg during pregnancy was higher than that in the overweight and obese group (70.24% vs. 42.86%, *p* < 0.01). Additionally, the fasting plasma glucose levels during OGTT in the overweight and obese group were significantly higher than those in the underweight and normal weight group (median glucose 4.93 mmol/L, IQR [4.54–5.23] mmol/L, vs. median glucose 4.56 mmol/L, IQR [4.26–4.87] mmol/L, *p* < 0.01). No statistically significant differences were observed between the two groups in terms of maternal age at delivery, parity, level of weight gain during pregnancy, gestational age at birth, plasma glucose levels at 60 and 120 min during OGTT, neonatal birth weight, height, or the proportions of preterm and macrosomic infants.

**Table 1:** The maternal and offspring characteristics during pregnancy and the perinatal period in the overweight and obese group and the underweight and normal weight group.

**Characteristics**	**Underweight and normal weight group (** **n** = 84**)**	**Overweight and obese group (** **n** = 28**)**	**Statistic**	**p**
Maternal age (years)	31.00 (29.00, 33.25)	32.00 (29.75, 34.00)	−0.99	0.32
Age ≥ 35 years, *n* (%)	9 (10.71)	6 (21.43)	1.26	0.26
Parity, *n* (%)				
Primipara	45 (53.57)	15 (53.57)	0.00	1.00
Multipara	39 (46.43)	13 (46.43)		
Gestational weight gain (kg)	12.50 (10.00, 15.00)	10.00 (6.12, 15.00)	−1.57	0.12
Gestational weight gain ≥ 11 kg (%)	59 (70.24)	12 (42.86)	6.78	< 0.01 ^∗^
Gestational age (weeks)	39.00 (38.00, 39.00)	39.00 (37.75, 39.00)	−0.69	0.49
OGTT results during pregnancy				
Fasting plasma glucose (mmol/L)	4.56 (4.26, 4.87)	4.93 (4.54, 5.23)	−2.67	< 0.01 ^∗^
1‐h plasma glucose (mmol/L)	7.89 (6.57, 9.76)	8.30 (7.11, 9.05)	−0.33	0.74
2‐h plasma glucose (mmol/L)	7.00 (6.04, 8.83)	6.87 (6.17, 8.02)	−0.41	0.68
GDM, *n* (%)	39 (46.43)	14 (50.00)	0.11	0.74
Infant				
Birth weight (kg)	3.21 (2.96, 3.54)	3.40 (3.00, 3.71)	−0.65	0.52
Birth height (cm)	50.00 (49.00, 51.00)	50.50 (48.75, 51.00)	−0.58	0.56
Preterm birth, *n* (%)	7 (8.33)	3 (10.71)	0.00	1.00
Fetal macrosomia, *n* (%)	3 (3.57)	3 (10.71)	0.94	0.33

*Note:* Statistics: maternal age, gestational weight gain, gestational weeks, fasting plasma glucose, 1‐h plasma glucose, 2‐h plasma glucose, birth weight, and height for Mann–Whitney *U* test; age ≥ 35 years, parity, gestational weight gain ≥ 11 kg, GDM, preterm birth, and fetal macrosomia for chi‐square test or Fisher test. Continuous data are expressed as median (IQR). Categorical data are expressed as numbers (percentages) of cases.

Abbreviations: GDM, gestational diabetes mellitus; OGTT, oral glucose tolerance test.

^∗^ *p* < 0.01.

Table 2 presents the maternal general and metabolic indices 4–7 years postpartum in the overweight and obese group and the underweight and normal weight group. The maternal BMI and the proportion of overweight and obese individuals were higher in the overweight and obese group compared to the underweight and normal weight group during this period. Additionally, the overweight and obese group demonstrated higher indices such as WHR, *C*‐index, VAI, LAP, TSF, and body fat percentage than the underweight and normal weight group. With regard to glucose and lipid metabolism, fasting insulin, HOMA‐IR, and fasting and 120‐min C‐peptide levels were elevated in the overweight and obese group compared to the underweight and normal weight group. However, no significant differences were observed between the two groups in fasting and 2‐h postprandial glucose levels during an OGTT, glycated hemoglobin (HbA1c) levels, or the prevalence of impaired fasting glucose (IFG) or impaired glucose tolerance (IGT) and diabetes mellitus (DM). The overweight and obese group had higher levels of TC, TG, LDL‐C, TC/HDL‐C, sd‐LDL, Apo*β*, lipoprotein(a), while HDL levels and lipoprotein(a) were lower in the overweight and obese group compared to the underweight and normal weight group. No significant difference was noted in ApoA1 and FFA between the groups. Differences in TNF‐*α* levels were evident between the two maternal groups, whereas no differences were found in other inflammatory factors, including IL‐6, IL‐8, IL‐2, IL‐1*β*, TNF‐*β*, and GDF15 levels. Furthermore, no significant difference was observed in the levels of adipocytokines, specifically adiponectin and leptin, between the overweight and obese group and the underweight and normal weight group.

**Table 2:** The maternal general and metabolic indices 4–7 years postpartum in the overweight and obese group and the underweight and normal weight group.

**Variables**	**Underweight and normal weight group (** **n** = 84**)**	**Overweight and obese group (** **n** = 28**)**	**Statistic**	**p**
General indices				
Systolic blood pressure (mmHg)	110.00 (101.75, 118.00)	113.50 (110.00, 120.00)	−1.94	0.05
Diastolic blood pressure (mmHg)	70.00 (62.75, 74.00)	70.00 (66.50, 76.50)	−1.56	0.12
Postpartum BMI (kg/m^2^)	21.92 (20.31, 23.12)	26.51 (24.84, 28.96)	−6.45	< 0.01 ^∗^
Postpartum weight gain, *n* (%)	62 (73.81)	17 (60.71)	1.73	0.19
Postpartum overweight or obese percentage			53.10	< 0.01 ^∗^
Underweight and normal weight, *n* (%)	73 (86.90)	6 (21.43)		
Overweight, *n* (%)	11 (13.10)	11 (39.29)		
Obesity, *n* (%)	0 (0.00)	11 (39.29)		
WHR	0.82 (0.79, 0.85)	0.89 (0.82, 0.90)	−3.36	< 0.01 ^∗^
*C*‐index	1.17 (1.13, 1.23)	1.23 (1.17, 1.26)	−2.03	0.04 ^∗^
VAI	1.09 (0.82, 1.71)	2.27 (1.52, 2.79)	−3.89	< 0.01 ^∗^
LAP	15.41 (10.47, 22.78)	40.48 (27.74, 57.24)	−5.01	< 0.01 ^∗^
TSF (mm)	13.15 (11.43, 15.00)	17.15 (15.40, 19.77)	−5.08	< 0.01 ^∗^
Body fat percentage	31.45 (25.80, 34.32)	37.25 (34.10, 40.60)	−5.51	< 0.01 ^∗^
Metabolic indices				
HbA1c (%)	5.50 (5.30, 5.70)	5.55 (5.38, 5.90)	−1.40	0.16
OGTT results 4–7 years postpartum				
Fasting plasma glucose (mmol/L)	5.20 (4.90, 5.50)	5.30 (4.86, 5.82)	−0.95	0.34
2‐h plasma glucose (mmol/L)	6.35 (5.40, 7.73)	7.00 (5.77, 8.88)	−1.59	0.11
Fasting insulin (uU/mL)	9.04 (6.21, 12.20)	11.59 (8.48, 22.15)	−2.47	0.01 ^∗^
2‐h insulin (uU/mL)	49.98 (33.24, 65.13)	62.33 (32.44, 96.62)	−1.94	0.05
HOMA‐IR	2.06 (1.46, 2.99)	2.80 (1.69, 6.39)	−2.58	< 0.01 ^∗^
Fasting C‐peptide (ng/ml)	2.00 (1.60, 2.50)	2.82 (2.07, 3.54)	−3.74	< 0.01 ^∗^
2‐h C‐peptide (ng/ml)	8.17 (6.37, 9.53)	9.60 (7.48, 12.31)	−2.08	0.04 ^∗^
Postpartum glycemic status				
IFG or IGT, *n* (%)	22 (26.19)	8 (28.57)	4.04	0.13
DM, *n* (%)	5 (5.95)	5 (17.86)		
TC (mmol/L)	4.56 (4.01, 5.02)	4.93 (4.16, 5.57)	−2.08	0.05 ^∗^
TG (mmol/L)	0.84 (0.60, 1.18)	1.41 (1.04, 1.71)	−3.68	< 0.01 ^∗^
HDL‐C (mmol/L)	1.40 (1.21, 1.61)	1.23 (1.05, 1.36)	−2.67	< 0.01 ^∗^
LDL‐C (mmol/L)	2.75 (2.36, 3.15)	3.15 (2.74, 3.61)	−2.40	0.02 ^∗^
TC/HDL‐C	3.31 (2.77, 3.73)	3.98 (3.46, 4.26)	−3.73	< 0.01 ^∗^
sd‐LDL (mmol/L)	0.77 (0.58, 0.94)	1.04 (0.88, 1.35)	−3.62	< 0.01 ^∗^
ApoA1 (mg/dL)	141.00 (128.00, 154.00)	138.00 (123.50, 147.25)	−1.17	0.24
Apo*β* (mg/dL)	89.00 (78.00, 108.00)	109.50 (94.75, 119.00)	−3.36	< 0.01 ^∗^
Lipoprotein(a) (mg/L)	92.00 (44.50, 182.50)	51.50 (35.75, 84.00)	−2.45	0.01 ^∗^
FFA (*μ*Eq/L)	410.00 (260.00, 566.50)	436.50 (352.25, 607.25)	−1.20	0.23
Adiponectin (ng/mL)	2787.73 (1889.57, 3603.91)	2453.39 (1579.42, 2875.85)	−1.96	0.05
Leptin (pg/mL)	6850.84 (5913.24, 9403.80)	8827.22 (7295.87, 9981.60)	−1.76	0.08

*Note:* Statistics: systolic blood pressure, diastolic blood pressure, postpartum BMI, WHR, *C*‐index, VAI, LAP, TSF, body fat percentage, blood glucose, blood lipids, inflammatory markers, and leptin for Mann–Whitney *U* test; postpartum weight gain, postpartum overweight or obese percentage, and postpartum glycemic status for chi‐square test or Fisher test. Continuous data are expressed as median (interquartile range). Categorical data are expressed as numbers (percentages) of cases.

Abbreviations: Apo*β*, apolipoprotein *β*; ApoA1, apolipoprotein A1; BMI, body mass index; *C*‐index, conicity index; DM, diabetes mellitus; FFA, free fatty acids; HbA1c, glycated hemoglobin A1c; HDL‐C, high‐density lipoprotein cholesterol; HOMA‐IR, homeostasis model assessment of insulin resistance; IFG, impaired fasting glucose; IGT, impaired glucose tolerance; LAP, lipid accumulation product; LDL‐C, low‐density lipoprotein cholesterol; OGTT, oral glucose tolerance test; sd‐LDL, small dense low‐density lipoprotein; TC, total cholesterol; TC/HDL‐C, total cholesterol to high‐density lipoprotein cholesterol ratio; TG, triglycerides; TSF, triceps skinfold thickness; VAI, visceral adiposity index; WHR, waist‐to‐hip ratio.

^∗^ *p* < 0.05.

Table 3 presents the anthropometric and metabolic factors of the offspring aged 4–7 years. The offspring of mothers in the overweight and obese group exhibited significantly higher TSF than those in the underweight and normal weight group (median: 8.25 mm, IQR: 6.38–10.77 vs. median: 6.60 mm, IQR: 5.70–8.93; *p* < 0.01). However, no statistically significant differences were detected between the two groups in age, sex distribution, blood pressure, BMI, the proportion classified as overweight or obese, or WHR, with both blood pressure [17] and weight status [12] defined using the age‐ and sex‐specific reference standards detailed in Supporting Information 1: Table S1.

**Table 3:** The anthropometric factors of offspring aged 4–7 years in the overweight and obese group and the underweight and normal weight group.

**Variables**	**Underweight and normal weight group (** **n** = 84**)**	**Overweight and obese group (** **n** = 28**)**	**Statistic**	**p**
Offspring age (years)	5.75 (5.00, 6.00)	5.50 (5.00, 6.00)	−0.68	0.50
Offspring gender, *n* (%)			2.04	0.15
Boys	40 (47.62)	9 (32.14)		
Girls	44 (52.38)	19 (67.86)		
Systolic blood pressure (mmHg)	96.00 (90.75, 101.00)	98.00 (92.00, 102.25)	−1.35	0.18
Diastolic blood pressure (mmHg)	61.00 (57.00, 63.00)	60.50 (58.00, 63.50)	−0.24	0.81
Offspring BMI (kg/m^2^)	15.29 (14.37, 16.64)	15.75 (14.91, 17.08)	−1.39	0.16
Offspring overweight or obesity percentage			0.76	0.252
Underweight and normal weight, *n* (%)	64 (76.19)	19 (67.86)		
Overweight, *n* (%)	14 (16.67)	4 (14.29)		
Obesity, *n* (%)	6 (7.14)	5 (17.86)		
Offspring WHR	0.89 (0.85, 0.92)	0.90 (0.87, 0.92)	−0.37	0.71
Offspring TSF (mm)	6.60 (5.70, 8.93)	8.25 (6.38, 10.77)	−2.31	0.05 ^∗^

*Note:* Statistics: Offspring age, systolic blood pressure, diastolic blood pressure, offspring BMI, offspring WHR, and offspring TSF for Mann–Whitney *U* test; offspring gender and offspring overweight or obese percentage for chi‐square test or Fisher′s test. Continuous data are expressed as median (interquartile range). Categorical data are expressed as numbers (percentages) of cases.

Abbreviations: BMI, body mass index; TSF, triceps skinfold thickness; WHR, waist‐to‐hip ratio.

^∗^ *p* < 0.05.

Univariate linear regression analysis revealed that prepregnancy BMI was associated with gestational age, gestational weeks, GDM, GWG, and postpartum indicators at 4–7 years, including BMI, WHR, VAI, LAP, TSF, HOMA‐IR, and blood lipid parameters, as well as offspring indicators at 4–7 years, such as BMI and TSF (Table 4). However, multiple linear regression analysis demonstrated that after adjusting for gestational age, parity, gestational weeks, and GDM, prepregnancy BMI was only significantly associated with GWG (*β* = −0.13, *p* = 0.017) and maternal BMI at 4–7 years postpartum (*β* = 0.78, *p* < 0.001), respectively. The mediating role of GWG in the association between prepregnancy BMI and offspring BMI was formally tested. The results indicated a statistically nonsignificant average causal mediation effect (ACME = 0.006, 95% CI: −0.062 to 0.12; *p* = 0.907). The average direct effect (ADE = 0.179, 95% CI: −0.006 to 0.38; *p* = 0.063) and the total effect (0.185, 95% CI: −0.02 to 0.45; *p* = 0.104) were also nonsignificant.

**Table 4:** Univariate and multiple regression analyses of the association between prepregnancy BMI and maternal general indices, metabolic indices, and anthropometric indices of offspring.

**Index**	**Univariate linear regression analysis**		**Multiple linear regression analysis**	
	**β** **95% CI**	**p**	**β** **95% CI**	**p**
Maternal age (years)	0.17 (0.01~0.33)	0.041 ^∗^	0.00 (−0.13 to approximately 0.13)	0.963
Parity				
Primipara	0.00 (reference)		0.00 (reference)	
Multipara	−0.19 (−1.37 to approximately 0.99)	0.754	−0.28 (−1.09 to approximately 0.53)	0.504
Gestational age (weeks)	−0.40 (−0.70 to approximately −0.09)	0.012 ^∗^	−0.35 (−0.60 to approximately −0.10)	0.007 ^∗^
GDM	1.21 (0.05~2.37)	0.043 ^∗^	0.68 (−0.29 to approximately 1.65)	0.174
Gestational weight gain (kg)	−0.17 (−0.29 to approximately −0.05)	0.007 ^∗^	−0.13 (−0.23 to approximately −0.02)	0.017 ^∗^
Birth weight (kg)	0.34 (−0.88 to approximately 1.56)	0.587	1.07 (0.01~2.13)	0.052
Postpartum BMI (kg/m^2^)	0.74 (0.64~0.84)	< 0.001 ^∗^	0.78 (0.53~1.03)	< 0.001 ^∗^
WHR	16.85 (7.36~26.33)	< 0.001 ^∗^	−3.31 (−15.05 to approximately 8.44)	0.583
*C*‐index	4.61 (−2.88 to approximately 12.10)	0.230	−3.79 (−13.19 to approximately 5.62)	0.432
VAI	0.47 (0.22~0.72)	< 0.001 ^∗^	0.58 (−0.82 to approximately 1.98)	0.416
LAP	0.04 (0.03~0.06)	< 0.001 ^∗^	0.02 (−0.06 to approximately 0.11)	0.581
TSF (mm)	0.38 (0.25~0.51)	< 0.001 ^∗^	−0.02 (−0.14 to approximately 0.10)	0.725
HOMA‐IR	0.54 (0.36~0.72)	< 0.001 ^∗^	−0.24 (−0.50 to approximately 0.01)	0.065
TC (mmol/L)	0.79 (0.09~1.48)	0.029 ^∗^	2.45 (−1.10 to approximately 6.00)	0.179
TG (mmol/L)	1.04 (0.49~1.58)	< 0.001 ^∗^	−1.98 (−4.48 to approximately 0.52)	0.124
HDL‐C (mmol/L)	−3.02 (−5.01 to approximately −1.03)	0.004 ^∗^	−3.87 (−10.12 to approximately 2.38)	0.228
LDL‐C (mmol/L)	0.95 (0.15~1.74)	0.022 ^∗^	−1.32 (−4.58 to approximately 1.94)	0.430
TC/HDL‐C	1.15 (0.59~1.71)	< 0.001 ^∗^	−1.58 (−3.47 to approximately 0.32)	0.106
sd‐LDL (mmol/L)	3.44 (1.98~4.91)	< 0.001 ^∗^	1.73 (−0.46 to approximately 3.92)	0.124
ApoA1 (mg/dL)	−0.01 (−0.02 to approximately 0.01)	0.405	−0.00 (−0.01 to approximately 0.01)	0.718
Apo*β* (mg/dL)	0.04 (0.02~0.06)	0.002 ^∗^	−0.01 (−0.07 to approximately 0.04)	0.681
Offspring BMI (kg/m^2^)	0.26 (0.04~0.47)	0.020 ^∗^	−0.10 (−0.26 to approximately 0.07)	0.257
Offspring WHR	−3.42 (−12.72 to approximately 5.88)	0.472	−3.49 (−9.23 to approximately 2.24)	0.236
Offspring TSF (mm)	0.35 (0.12~0.58)	0.004 ^∗^	0.08 (−0.11 to approximately 0.28)	0.399

Abbreviations: Apo*β*, apolipoprotein *β*; ApoA1, apolipoprotein A1; BMI, body mass index; *C*‐index, conicity index; GDM, gestational diabetes mellitus; HDL‐C, high‐density lipoprotein cholesterol; HOMA‐IR, homeostasis model assessment of insulin resistance; LAP, lipid accumulation product; LDL‐C, low‐density lipoprotein cholesterol; sd‐LDL, small dense low‐density lipoprotein; TC, total cholesterol; TC/HDL‐C, total cholesterol to high‐density lipoprotein cholesterol ratio; TG, triglycerides; TSF, triceps skinfold thickness; VAI, visceral adiposity index; WHR, waist‐to‐hip ratio.

^∗^ *p* < 0.05.

As presented in Tables 5 and 6, after adjusting for parity, gestational age, GWG, gestational weeks, and GDM, logistic regression analysis indicated that prepregnancy overweight and obesity was a significant risk factor for maternal overweight and obesity 4–7 years postpartum (OR 30.70, 95% CI [8.69–108.51], *p* < 0.01) but was not a significant risk factor for overweight and obesity in offspring aged 4–7 years (OR 1.49, 95% CI [0.54–4.06], *p* = 0.440).

**Table 5:** Logistic regression analyses of the association between maternal overweight and obesity 4–7 years postpartum and prepregnancy overweight and obesity.

	**Model 1**		**Model 2**	
	**OR (95% CI)**	**p**	**OR (95% CI)**	**p**
Prepregnancy underweight and normal weight	1.00 (reference)		1.00 (reference)	
Prepregnancy overweight and obesity	24.33 (8.08~73.32)	< 0.01 ^∗^	30.70 (8.69~108.51)	< 0.01 ^∗^
GDM	1.07 (0.47~2.41)	0.87	0.99 (0.26~3.77)	0.99
Parity				
Primipara	1.00 (reference)		1.00 (reference)	
Multipara	0.95 (0.42~2.14)	0.89	0.74 (0.24~2.34)	0.61
Maternal age	1.12 (0.99~1.26)	0.06	1.14 (0.95~1.37)	0.16
Gestational weight gain	0.97 (0.89~1.06)	0.50	1.09 (0.96~1.24)	0.20
Gestational age	0.83 (0.67~1.04)	0.10	0.84 (0.59~1.18)	0.31

*Note:* Model 2: adjusted for parity, gestational age, gestational weight gain, and GDM.

Abbreviation: GDM, gestational diabetes mellitus.

^∗^ *p* < 0.01.

**Table 6:** Logistic regression analyses of the association between obesity and overweight in offspring aged 4–7 years and prepregnancy overweight and obesity.

	**Model 1**		**Model 2**	
	**OR (95% CI)**	**p**	**OR (95% CI)**	**p**
Prepregnancy underweight and normal weight	1.00 (reference)		1.00 (reference)	
Prepregnancy overweight and obesity	1.52 (0.59~3.88)	0.385	1.49 (0.54~4.06)	0.440
GDM	1.27 (0.54~2.96)	0.582	1.15 (0.41~3.24)	0.789
Parity				
Primipara	1.00 (reference)		1.00 (reference)	
Multipara	0.92 (0.39~2.14)	0.841	0.81 (0.32~2.07)	0.667
Maternal age	1.03 (0.91~1.15)	0.665	1.04 (0.91~1.19)	0.590
Gestational weight gain	0.96 (0.88~1.05)	0.364	0.95 (0.85~1.07)	0.407
Gestational weeks	1.26 (0.92~1.72)	0.158	1.33 (0.96~1.86)	0.090

*Note:* Model 2: adjusted for parity, gestational age, gestational weight gain, and GDM.

Abbreviation: GDM, gestational diabetes mellitus.

## 4. Discussion

This study confirms a robust and independent association between prepregnancy overweight/obesity and long‐term maternal weight status. Specifically, mothers with prepregnancy overweight or obesity faced a substantially increased risk of remaining overweight or obese 4–7 years postpartum, even after adjusting for key obstetric and metabolic factors. This underscores that prepregnancy overweight is not merely a transient condition but a major predictor of sustained weight retention, shifting the clinical focus from short‐term postpartum weight status to long‐term weight trajectory. This finding reinforces the consistent signal from existing literature that prepregnancy BMI is a key determinant of postpartum weight outcomes, as supported by longitudinal cohorts [8] and studies reporting higher obesity prevalence among previously overweight women [16]. However, the novel contribution of our study lies in its extended follow‐up period of 4–7 years postpartum. Much of the existing literature, including influential reviews emphasizing considerable individual variability in weight retention [18], concentrates on the first 1–2 years postpartum—a period of acute physiological and behavioral transition. This longer‐term perspective helps reconcile seemingly inconsistent findings in the literature. For instance, while research indicates that excessive GWG is a strong predictor of retention [19], our study and others with longer follow‐up suggest that the influence of prepregnancy BMI may become increasingly independent of GWG over time. Thus, the risk related to prepregnancy overweight persists well beyond the early postpartum transition. This distinction is not merely chronological but points to a potential shift in underlying drivers: Whereas early postpartum weight may be strongly shaped by pregnancy‐related adaptations and GWG, long‐term weight status appears to be more firmly anchored in prepregnancy metabolic and behavioral predispositions.

Our analysis did not establish a significant association between maternal prepregnancy BMI and offspring weight status at age 4–7 years. This result diverges from a body of literature that often identifies such a link, as noted below, but finds context in other relevant studies. For instance, our null finding is consistent with research reporting no direct association between maternal prepregnancy BMI and newborn birth weight [20], although that study was limited to the birth period. More importantly, it may be explained by the influence of other, potentially more dominant, intrauterine pathways. Research indicates that fetal exposure to elevated maternal glucose levels can independently increase offspring obesity risk by age 3 [21], suggesting that specific metabolic exposures might supersede the influence of prepregnancy BMI on early childhood weight in some populations. This highlights the multifactorial nature of the developmental origins of obesity, where factors such as maternal glycemia, birth weight, and postnatal diet interact to shape risk. Converging evidence from recent studies underscores a significant association between maternal prepregnancy overweight/obesity and an elevated risk of offspring overweight/obesity. While one study [10] reported this link as most pronounced in children aged 2–5 years, others [22–24] found it to be particularly strong at 6–7 years of age within a broader 4–8‐year‐old cohort. These findings collectively establish prepregnancy maternal weight as a key determinant of early childhood weight outcomes. While previous research consistently links maternal prepregnancy overweight/obesity with offspring weight outcomes, a recent study did not replicate these findings. Several factors may explain this discrepancy. First, the age at follow‐up varied across studies, and the association may be age‐specific. Second, the relatively modest sample size may have limited statistical power to detect subtle effects. Additionally, contrary to prior reports, this study found no significant association between GWG and offspring overweight and obesity—a divergence potentially influenced by regional differences in gestational weight management practices. Furthermore, unlike previous studies [25, 26], this study found no significant association between GDM and overweight/obesity in mothers or offspring 4–7 years postdelivery (Supporting Information 2: Table S2). These results highlight the complexity of the relationship between GDM and weight status, warranting further investigation.

Maternal prepregnancy BMI is strongly associated with offspring birth weight, primarily due to intrauterine fetal programming. This mechanism promotes higher birth weight and sustained offspring growth, independent of lifestyle or nutritional factors. Optimal maternal nutrition supports fetal development by ensuring nutrient supply and regulating metabolic pathways, including the hypothalamic–pituitary axis, pancreatic islets, and adipose tissue. Maternal overweight and obesity alter this programming, leading to larger neonates [27]. Both maternal overweight and obesity lead to larger neonates due to alterations in fetal metabolic programming, particularly affecting the hypothalamic–pituitary axis, pancreatic islet cells, and adipose tissue. These alterations set the stage for offspring overweight and obesity later in life [28]. The developmental overnutrition hypothesis [29] explains that maternal obesity, insulin resistance, and excessive GWG elevate maternal glucose and TG. These metabolites cross the placenta, increasing fetal nutrient availability. This stimulates fetal insulin secretion, promoting accelerated growth and higher birth weight [23].

This study examined associations between prepregnancy BMI and metabolic markers. Women with prepregnancy overweight/obesity had higher fasting glucose during pregnancy. At 4–7 years postpartum, this group exhibited greater insulin resistance and consistently adverse lipid profiles, including elevated TG, low‐density lipoprotein (LDL), TC/HDL ratio, sd‐LDL, and apolipoprotein B. These findings align with and extend previous longitudinal research. For example, one study reported that postpartum women with substantial weight retention had adverse changes in lipid profiles and insulin sensitivity by Year 3, with graded worsening across metabolic indices by Year 5 [30]. Another study identified prepregnancy BMI as a significant correlate of abnormal lipid profiles during pregnancy [31]. Our results further support the link between prepregnancy BMI and persistent dyslipidemia postpartum. However, in contrast to some prior reports, we did not detect significant associations between prepregnancy BMI and certain glycemic indices after adjustment for confounders. This discrepancy may be explained by differences in population characteristics, follow‐up duration, or measurement protocols. Importantly, recent lipidomics research has revealed that prepregnancy obesity is associated with specific alterations in phospholipid and sphingomyelin levels during pregnancy [32], suggesting that traditional lipid measures may not fully capture the subtle metabolic disturbances related to prepregnancy BMI. Thus, while our study confirms the strong and lasting influence of prepregnancy BMI on postpartum lipid profiles, its relationship with glucose metabolism appears more variable and may be modulated by factors such as lifestyle changes, weight trajectory after delivery, or underlying genetic susceptibility.

### 4.1. Strengths

The study stands out for its thorough postpartum monitoring spanning 4–7 years, offering valuable insights into the lasting effects of prepregnancy overweight and obesity on mothers and children. In addition to analyzing maternal weight outcomes, it delves into the impact on offspring, uncovering potential intergenerational repercussions. Moreover, the research investigates the association between prepregnancy BMI and a range of postpartum factors, including glucose and lipid metabolism, inflammatory markers, and abdominal obesity indicators, providing a nuanced perspective on the broader implications. This comprehensive approach sheds light on the complexities of obesity in pregnancy and its aftermath, contributing to a more holistic understanding of its consequences.

### 4.2. Limitations

This study has several limitations that should be considered when interpreting the findings. First, the relatively modest sample size may have reduced statistical power, increasing the risk of Type II error. This is a plausible explanation for why some associations—particularly those involving offspring outcomes or more complex mediating pathways—did not reach statistical significance, even when point estimates suggested a trend. Consequently, our null findings in these areas should be interpreted with caution.

Second, the absence of data on key postnatal lifestyle factors (e.g., breastfeeding duration, childhood diet, and physical activity levels) represents a significant constraint. These unmeasured variables are potential confounders or mediators of the observed associations. For example, the link between maternal prepregnancy BMI and offspring weight could be partly explained by shared family eating habits rather than a direct intrauterine effect. Therefore, while our models adjusted for major obstetric factors, residual confounding by lifestyle remains possible, potentially biasing our effect estimates upward.

Third, the application of Chinese‐specific BMI cut‐off points, while clinically appropriate for our population, may limit direct comparability with studies using WHO criteria. This choice likely results in a higher estimated prevalence of overweight/obesity in our sample, which should be considered when comparing our prevalence rates to international literature. However, it does not affect the internal validity of the associations reported between BMI categories and other outcomes.

Notwithstanding these limitations, our findings underscore critical public health implications. The persistent link between prepregnancy and long‐term postpartum weight status reinforces the need for a shift toward proactive, preconception health strategies. Healthcare systems should prioritize enabling young women to access counseling on nutrition and healthy lifestyles well before pregnancy planning, as emphasized in recent frameworks [33, 34]. Moreover, effective interventions must adopt a family‐centered approach, recognizing that the child′s diet and lifestyle are shaped by the entire household environment, not solely by maternal factors.

### 4.3. Conclusion

This cohort study demonstrated an association between prepregnancy overweight/obesity and persistent maternal weight retention at 4–7 years postpartum. These findings underscore that prepregnancy weight status is a strong predictor of long‐term maternal health trajectory, extending beyond the immediate postpartum period. This association thus reinforces the critical importance of preconception weight management as a vital preventive health strategy.

## Disclosure

All authors critically reviewed and approved the final version of the manuscript, contributing to its overall quality and coherence.

## Conflicts of Interest

The authors declare no conflicts of interest.

## Author Contributions

X.Z. and N.Y. conceived the research concept, designed the study, and collaboratively wrote the manuscript. X.Z., N.Y., and D.Z. were responsible for recruiting patients and collecting the necessary samples. Additionally, D.Z. and X.X. contributed significantly to the data acquisition process. X.Z., N.Y., and D.Z. jointly undertook the tasks of data collection, analysis, and manuscript revision, ensuring the accuracy and integrity of the results. N.Y. and D.Z. contributed equally to this work and should be considered co‐first authors.

## Funding

The study was supported by the Beijing Municipal Science & Technology Commission (Z221100007422114).

## Supporting Information

Additional supporting information can be found online in the Supporting Information section.

## Supporting information


**Supporting Information 1** Table S1. Reference cut‐off points for children aged 4–7 years: BMI and normal blood pressure.

**Supporting Information 2** Table S2. The proportion of overweight and obesity in mothers and their offspring 4–7 years postpartum between groups with and without GDM.

## Data Availability

The data that support the findings of this study are available from the corresponding author upon reasonable request.
