# Supplementary material for: Maternal Prepregnancy Overweight: Associations With Maternal and Offspring Weight 4–7 Years Postpartum
Source: J Diabetes Res. 2026 Feb 5;2026:9989579. doi: 10.1155/jdr/9989579 (PMC12877321; doi:10.1155/jdr/9989579)
Supplement: Supplementary file 1 — Supporting Information 1 Table S1. Reference cut‐off points for children aged 4–7 years: BMI and normal blood pressure. [file JDR-2026-9989579-s002.docx]

| Age(years) | Boys | | Girls | |
| --- | --- | --- | --- | --- |
|  | Overweight | Obesity | Overweight | Obesity |
| 4.0 | 16.5 | 17.8 | 16.7 | 18.1 |
| 4.5 | 16.4 | 17.8 | 16.6 | 18.1 |
| 5.0 | 16.5 | 17.9 | 16.6 | 18.2 |
| 5.5 | 16.6 | 18.1 | 16.7 | 18.3 |
| 6.0 | 16.8 | 18.4 | 16.7 | 18.4 |
| 6.5 | 17.0 | 18.8 | 16.8 | 18.6 |
| 7.0 | 17.2 | 19.2 | 16.9 | 18.8 |

Supplementary Table 1. Reference cut-off points for children aged 4–7 years: body mass index (BMI) and normal blood pressure

Table 1a. BMI cut-off points for screening overweight and obesity in children aged 4–7 years (kg/m²)^[12]^

Table 1b. Normal blood pressure cut-off points (Systolic blood pressure/Diastolic blood pressure < 90th percentile for age, sex, and height) for children aged 4–7 years (mmHg)^[14]^

| Age(years) | BP Percentile | Boys | | | | | | Girls | | | | | |
| --- | --- | --- | --- | --- | --- | --- | --- | --- | --- | --- | --- | --- | --- |
|  |  | Systolic blood pressure | | | Diastolic blood pressure | | | Systolic blood pressure | | | Diastolic blood pressure | | |
|  |  | Height Percentile or Measured Height | | | Height Percentile or Measured Height | | | Height Percentile or Measured Height | | | Height Percentile or Measured Height | | |
|  |  | 5% | 50% | 95% | 5% | 50% | 95% | 5% | 50% | 95% | 5% | 50% | 95% |
| 4.0 | Height(cm) | 98.5 | 105.9 | 113.2 | 98.5 | 105.9 | 113.2 | 97.2 | 104.5 | 112.2 | 97.2 | 104.5 | 112.2 |
|  | 90^th^ | 102 | 105 | 107 | 60 | 62 | 64 | 103 | 106 | 108 | 62 | 65 | 67 |
| 5.0 | Height(cm) | 104.4 | 112.4 | 120.3 | 104.4 | 112.4 | 120.3 | 103.6 | 111.5 | 120.0 | 103.6 | 111.5 | 120.0 |
|  | 90^th^ | 103 | 106 | 108 | 63 | 65 | 67 | 104 | 107 | 110 | 64 | 67 | 70 |
| 6.0 | Height(cm) | 110.3 | 118.9 | 127.5 | 110.3 | 118.9 | 127.5 | 110.0 | 118.4 | 127.7 | 110.0 | 118.4 | 127.7 |
|  | 90^th^ | 105 | 107 | 110 | 66 | 68 | 69 | 105 | 108 | 111 | 67 | 69 | 71 |
| 7.0 | Height(cm) | 116.1 | 125.1 | 134.5 | 116.1 | 125.1 | 134.5 | 115.9 | 124.9 | 134.7 | 115.9 | 124.9 | 134.7 |
|  | 90^th^ | 106 | 109 | 111 | 68 | 70 | 71 | 106 | 109 | 112 | 68 | 70 | 72 |

The 50th, 90th, and 95th percentiles were derived by usingquantile regression on the basis of normal-weight children (BMI <85th percentile).
