# Supplementary material for: Maternal Prepregnancy Overweight: Associations With Maternal and Offspring Weight 4–7 Years Postpartum
Source: J Diabetes Res. 2026 Feb 5;2026:9989579. doi: 10.1155/jdr/9989579 (PMC12877321; doi:10.1155/jdr/9989579)
Supplement: Supplementary file 2 — Supporting Information 2 Table S2. The proportion of overweight and obesity in mothers and their offspring 4–7 years postpartum between groups with and without GDM. [file JDR-2026-9989579-s001.docx]

Supplementary Table 2 The proportion of overweight and obesity in mothers and their offspring 4-7 years postpartum between groups with and without GDM

| Variables | GDM (n = 53) | Non-GDM(n = 59) | Statistic | *P* |
| --- | --- | --- | --- | --- |
| Postpartum overweight or  obese percentage |  |  | 0.998 | 0.607 |
| Overweight, n(%) | 12(22.6%) | 10(16.9%) |  |  |
| Obesity, n(%) | 4(7.5%) | 7(11.9%) |  |  |
| Offspring overweight or  obesity percentage |  |  | 2.229 | 0.328 |
| Overweight, n(%) | 11(20.7%) | 7(11.9%) |  |  |
| Obesity, n(%) | 4(7.5%) | 7(11.9%) |  |  |

GDM, gestational diabetes mellitus.
